# Taming the Cytokine Storm: Therapeutic Strategies for Post‐Acute Sequelae of SARS‐CoV‐2 Infection

**DOI:** 10.1002/hsr2.72174

**Published:** 2026-03-22

**Authors:** Emmanuel Ifeanyi Obeagu

**Affiliations:** ^1^ Department of Biomedical and Laboratory Science Africa University Mutare Zimbabwe

**Keywords:** chronic inflammation, cytokine storm, immunomodulation, post‐acute sequelae of SARS‐CoV‐2 infection, therapeutic targeting

## Abstract

**Background and Aim:**

Post‐acute sequelae of SARS‐CoV‐2 infection (PASC), commonly referred to as long COVID, has emerged as a significant global health concern, marked by persistent symptoms and chronic inflammation following recovery from acute COVID‐19. A central driver of PASC pathogenesis is the sustained cytokine storm—an exaggerated and prolonged pro‐inflammatory response that leads to ongoing tissue injury and multisystem dysfunction. This review aims to synthesize current knowledge on cytokine dysregulation in PASC and evaluate emerging therapeutic strategies targeting these immunopathological mechanisms.

**Methods:**

A narrative review methodology was employed, drawing from recent peer‐reviewed publications, clinical trial databases, and immunological studies published between 2020 and 2025. Articles focusing on cytokine profiles in PASC, immune reprogramming, and immunomodulatory therapies were included. Mechanistic studies, biomarker research, and translational trials involving corticosteroids, cytokine inhibitors, Janus kinase (JAK) inhibitors, and novel biologics were critically analyzed.

**Results:**

The literature reveals that elevated levels of IL‐6, IL‐1β, TNF‐α, and IFN‐γ persist in a subset of PASC patients, contributing to chronic systemic and organ‐specific inflammation. Emerging therapies—including IL‐6 and IL‐1 receptor antagonists, JAK inhibitors, and CNS‐penetrant anti‐inflammatory agents—demonstrate promise in modulating cytokine storms and improving clinical outcomes. Recent insights into cytokine profiling, trained immunity, and neuroimmune crosstalk suggest potential for precision‐based interventions tailored to distinct inflammatory phenotypes in PASC.

**Conclusion:**

Persistent cytokine dysregulation underlies the pathophysiology of PASC and offers actionable targets for therapeutic intervention. Immunomodulatory strategies, when guided by biomarker profiling and systems biology approaches, hold promise for mitigating long‐term complications of COVID‐19. Future research should prioritize personalized treatment algorithms to address the heterogeneity of PASC and enhance patient recovery.

## 1 Introduction

The COVID‐19 pandemic has left a substantial proportion of survivors with persistent, sometimes debilitating health issues collectively termed post‐acute sequelae of SARS‐CoV‐2 infection (PASC) [1]. PASC is an umbrella term encompassing a broad spectrum of post‐infectious complications, including new‐onset cardiovascular disease, pulmonary fibrosis, metabolic disturbances such as diabetes, neurocognitive dysfunction, and fatigue. Within this spectrum, Long COVID represents a specific syndrome characterized by prolonged fatigue, cognitive impairment, dyspnea, and multisystem involvement, but it is important to note that not all manifestations of PASC fall under Long COVID [2, 3]. While the precise mechanisms driving PASC remain under active investigation, persistent immune dysregulation and low‐grade inflammation are increasingly recognized as central contributors in many patients. Evidence suggests that in some cases, elevated levels of proinflammatory cytokines—such as interleukin‐6 (IL‐6), tumor necrosis factor‐alpha (TNF‐α), and Interferon‐gamma (IFN‐γ)—persist beyond acute infection, correlating with ongoing tissue injury and organ‐specific dysfunction [4]. These observations have led to the hypothesis that a residual or sustained cytokine storm, rather than a single acute hyperinflammatory episode, may contribute to prolonged symptomatology. However, it is critical to emphasize that the causal role of cytokine dysregulation in PASC is not definitively established, and multiple mechanisms—including viral antigen persistence, autoimmunity, endothelial activation, and metabolic dysregulation—likely interact in complex and patient‐specific ways [5, 6].

Recent molecular and omics‐based studies have begun to elucidate mechanistic underpinnings of these processes. For example, transcriptomic analysis of human heart tissue demonstrates SARS‐CoV‐2‐mediated activation of the TNF‐NF‐κB pathway in cardiomyocytes, linking persistent inflammation to post‐COVID cardiovascular sequelae [7, 8]. Similarly, multi‐omics investigations reveal mitochondrial dysfunction and compromised blood‐brain barrier (BBB) integrity, offering mechanistic insight into fatigue, neurocognitive deficits, and neurological manifestations in post‐COVID patients. Collectively, these data suggest that cytokine dysregulation in PASC is not a uniform process but rather involves organ‐specific pathways, immune cell dysfunction, and ongoing inflammatory signaling [9, 10]. Understanding these mechanisms is critical for developing targeted therapeutic strategies. Interventions aimed at modulating residual cytokine activity, restoring immune homeostasis, and mitigating tissue injury are under investigation, with the ultimate goal of reducing long‐term morbidity. However, the heterogeneity of PASC phenotypes, variability in cytokine profiles, and limited longitudinal data pose significant challenges to defining universally effective treatments. Therefore, a nuanced, mechanistic approach that considers individual patient profiles and organ‐specific pathology is essential for advancing PASC therapeutics [11, 12]. This review synthesizes current evidence on cytokine dysregulation in PASC, integrating molecular, cellular, and pathway‐level insights, and discusses emerging therapeutic strategies aimed at taming the residual inflammatory storm. By combining mechanistic understanding with clinical implications, we aim to provide a framework for precision‐based interventions that can address the complex and evolving landscape of post‐COVID sequelae.

## 2 Aim

This review aims to comprehensively examine the current understanding of cytokine storm pathophysiology in PASC and to critically evaluate emerging immunomodulatory therapeutic strategies for its management.

### 2.1 Methods

This narrative review was conducted to synthesize current evidence on cytokine dysregulation and therapeutic strategies in PASC. The methodology was designed to provide a comprehensive, structured, and mechanistically informed overview, while acknowledging the heterogeneity of existing studies and the evolving nature of the literature.

### 2.2 Literature Search Strategy

A systematic search of the PubMed, Scopus, and Web of Science databases was conducted for publications from January 2020 to December 2025. Search terms included combinations of:

- “Post‐Acute Sequelae of SARS‐CoV‐2 Infection” OR “PASC” OR “Long COVID”.
- “Cytokine” OR “Inflammation” OR “Immune dysregulation”.
- “Mechanism” OR “Pathway” OR “Omics”.
- “Therapy” OR “Treatment” OR “Intervention”.

Additional studies were identified through the reference lists of selected articles and review papers. Only peer‐reviewed publications in English were considered. Preprints were included selectively if they provided novel mechanistic or omics‐based data, such as transcriptomic or pathway‐level analyses relevant to PASC pathogenesis.

### 2.3 Inclusion Criteria

Articles were included if they:

1. Investigated immune or cytokine dysregulation in patients with PASC.
2. Reported mechanistic or molecular evidence, including pathway analysis, omics profiling, or cell‐specific studies.
3. Discussed therapeutic strategies targeting inflammation, immune dysregulation, or cytokine pathways in PASC.
4. Studies focusing exclusively on acute COVID‐19 without post‐acute follow‐up were included only when relevant mechanistic insights could be extrapolated cautiously to PASC.

### 2.4 Data Extraction and Synthesis

Key information extracted from each study included:

- Patient population characteristics and PASC phenotypes.
- Cytokine profiles and immune cell alterations.
- Molecular and pathway‐level evidence (e.g., TNF‐NF‐κB activation, mitochondrial dysfunction, and BBB integrity).
- Therapeutic interventions and reported outcomes.

Given the narrative nature of this review, a qualitative synthesis was performed. Studies were integrated thematically to construct a mechanistic framework linking cytokine dysregulation, molecular pathways, and therapeutic strategies, emphasizing areas of convergence and ongoing uncertainty.

## 3 Limitations of the Methodology

As a narrative review, this study does not follow the formal protocol of a systematic review or meta‐analysis. The heterogeneity of PASC phenotypes, variability in cytokine measurement methodologies, and limited longitudinal data restrict quantitative comparison. Nevertheless, this structured approach provides a mechanistically informed overview, highlights critical gaps in knowledge, and identifies potential targets for future research and therapeutic intervention.

## 4 Cytokines Implicated in PASC

The persistence of symptoms in PASC, commonly referred to as long COVID, is increasingly being attributed to a state of chronic, low‐grade inflammation. Central to this inflammatory milieu is a sustained dysregulation of cytokine signaling, with elevated levels of several pro‐inflammatory mediators well beyond the resolution of acute infection. The cytokines most consistently implicated in PASC include IL‐6, IL‐1β, TNF‐α, IFN‐γ, and IL‐8, among others [5]. IL‐6 has emerged as a key orchestrator of both acute COVID‐19 severity and chronic inflammatory processes in PASC. Persistently elevated IL‐6 levels have been linked to ongoing fatigue, mood disturbances, and neuroinflammation. This cytokine acts as a potent inducer of the hepatic acute‐phase response, influencing the synthesis of C‐reactive protein (CRP) and fibrinogen, and is known to disrupt BBB integrity, potentially contributing to the neurocognitive symptoms observed in long COVID [13].

IL‐1β is another cytokine that remains elevated in a subset of patients with PASC. A central mediator of the innate immune response, IL‐1β activates endothelial cells, recruits leukocytes, and induces fever. Its chronic elevation may exacerbate endothelial dysfunction, contributing to the cardiovascular manifestations of long COVID, including postural orthostatic tachycardia syndrome (POTS) and palpitations. IL‐1β also contributes to neuroinflammation and may play a role in the pathogenesis of cognitive impairment associated with PASC [14]. TNF‐α, a key effector of inflammation and apoptosis, has also been implicated in the long‐term sequelae of SARS‐CoV‐2 infection. Elevated TNF‐α can perpetuate a pro‐inflammatory feedback loop, maintaining immune activation and tissue injury. It has been associated with persistent pulmonary symptoms, including cough and dyspnea, potentially due to its role in promoting fibrosis and alveolar damage. Additionally, TNF‐α has been linked to muscle wasting and fatigue in chronic inflammatory states, which may underlie the profound weakness reported by many patients with PASC [15].

IFN‐γ, a type II interferon, plays a dual role in viral infections by mediating antiviral immunity and modulating immune cell activation. In acute COVID‐19, its early expression is crucial for viral clearance. However, in the context of PASC, prolonged IFN‐γ signaling can become maladaptive, sustaining macrophage activation and contributing to tissue damage. Persistent IFN‐γ activity has been associated with ongoing immune dysregulation and may contribute to both systemic symptoms and localized tissue dysfunction in organs such as the lungs and brain [16]. IL‐8, a chemokine with strong neutrophil‐attracting properties, is often elevated in patients with lingering respiratory symptoms. Its overexpression may reflect unresolved neutrophil‐driven inflammation in the lung parenchyma, contributing to dyspnea and exercise intolerance. Beyond pulmonary effects, IL‐8 may play a role in systemic vascular inflammation, increasing the risk of thromboembolic events in long COVID patients (Table 1) [17].

**Table 1:** Cytokines implicated in postacute sequelae of SARS‐CoV‐2 infection.

Cytokine	Primary source(s)	Role in PASC pathophysiology	Associated symptoms/outcomes
IL‐6	Macrophages, endothelial cells, T cells	Promotes systemic inflammation; activates hepatic acute‐phase response; disrupts blood‐brain barrier	Fatigue, neuroinflammation, cognitive dysfunction, myalgia
IL‐1β	Monocytes, macrophages	Induces fever and vascular inflammation; drives endothelial activation	Chest pain, cardiovascular symptoms, brain fog
TNF‐α	Macrophages, T cells	Promotes apoptosis, chronic inflammation, and fibrosis	Fatigue, muscle weakness, pulmonary fibrosis
IFN‐γ	NK cells, Th1 cells	Sustains antiviral state; promotes macrophage activation	Autoimmunity, delayed tissue recovery, systemic inflammation
IL‐8 (CXCL8)	Monocytes, epithelial cells	Recruits and activates neutrophils; promotes pulmonary inflammation	Dyspnea, cough, microvascular injury
IL‐10	Regulatory T cells, macrophages	Anti‐inflammatory feedback; may be insufficient to control hyperinflammation	Immune dysregulation, prolonged symptoms
CXCL10 (IP‐10)	Monocytes, endothelial cells	Attracts activated T cells; marker of interferon response	Lung injury, persistent immune activation
Granulocyte‐macrophage colony‐stimulating factor	T cells, macrophages	Enhances differentiation of pro‐inflammatory macrophages	Multiorgan inflammation, respiratory symptoms

## 5 Pathophysiology of Cytokine Storms in PASC

The cytokine storm in PASC represents a complex, dysregulated immune response that extends beyond the acute phase of COVID‐19, contributing to persistent inflammation and tissue damage. Unlike the transient hyperinflammation seen during acute infection, PASC‐associated cytokine storms often involve prolonged activation of innate and adaptive immune pathways, which can perpetuate chronic symptoms and multi‐organ involvement [5]. At the core of this dysregulated response is the sustained overproduction of pro‐inflammatory cytokines and chemokines, including IL‐6, TNF‐α, IL‐1β, IFN‐γ, and chemokines such as CCL2 and CXCL10. These mediators contribute to endothelial activation, vascular permeability, and recruitment of immune cells to various tissues, resulting in local and systemic inflammation. Persistent endothelial dysfunction may also promote a prothrombotic state, leading to microvascular injury and organ damage frequently reported in Long COVID patients [13, 14].

Several mechanisms have been proposed to explain the chronicity of cytokine dysregulation in PASC. One key factor is viral persistence or the presence of residual viral antigens in immune‐privileged sites, which continuously stimulate innate immune cells such as macrophages and dendritic cells. Additionally, maladaptive immune responses characterized by T‐cell exhaustion, aberrant B‐cell activation, and autoantibody production may further drive inflammation and tissue injury. Dysregulated interferon signaling and impaired resolution of inflammation also contribute to the sustained cytokine storm [15, 16]. Furthermore, genetic and environmental factors may influence individual susceptibility to prolonged cytokine dysregulation. Preexisting conditions such as metabolic syndrome, cardiovascular disease, and autoimmune disorders have been associated with heightened inflammatory responses and worse PASC outcomes. The interplay of these factors underscores the heterogeneity of PASC and highlights the need for personalized therapeutic approaches targeting specific immunopathological pathways.

## 6 Cytokine Profiling for Patient Stratification

A fundamental challenge in the management of PASC lies in its clinical heterogeneity. Patients may present with diverse and overlapping symptoms—ranging from fatigue and myalgia to neurocognitive dysfunction and cardiovascular abnormalities. Emerging evidence suggests that this heterogeneity is mirrored at the immunological level, particularly in cytokine expression profiles. Advanced immunophenotyping and multiplex cytokine assays have uncovered that subsets of PASC patients exhibit distinct cytokine signatures. For example, one group may display persistently elevated IL‐6 and TNF‐α, while others may present with heightened type I or type II interferon responses. These patterns not only reflect divergent immune responses but may also predict clinical phenotypes and therapeutic responsiveness. This evolving understanding calls for a shift toward personalized medicine in PASC—using cytokine profiling to identify dominant inflammatory pathways and tailor treatment strategies accordingly. Such stratification could maximize therapeutic efficacy while minimizing unnecessary immunosuppression [10].

## 7 Trained Immunity and Epigenetic Reprogramming

Traditionally, immunological memory was considered a feature exclusive to adaptive immunity. However, the concept of trained immunity—whereby innate immune cells undergo long‐term functional reprogramming following an initial stimulus—has challenged this dogma. In the context of PASC, increasing evidence suggests that monocytes and macrophages undergo epigenetic modifications following SARS‐CoV‐2 exposure, resulting in sustained hyperresponsiveness to subsequent challenges. This epigenetic reprogramming involves histone modifications and altered transcriptional landscapes that perpetuate the production of pro‐inflammatory cytokines like IL‐1β and IL‐6, even in the absence of active viral infection. The implications are profound: rather than persistent viral replication, it may be the maladaptive memory of the innate immune system that sustains inflammation in long COVID. Targeting these epigenetic alterations—through histone deacetylase inhibitors or BET (bromodomain and extraterminal domain) inhibitors—offers a novel therapeutic avenue. These agents have the potential to reset the inflammatory tone of innate immune cells, thereby restoring homeostasis without the need for broad immunosuppression [18, 19].

## 8 Neuroimmune Crosstalk and Microglial Priming

Neurocognitive and neuropsychiatric symptoms—including brain fog, memory disturbances, and anxiety—are among the most debilitating features of PASC. Recent studies have highlighted the critical role of neuroimmune interactions in perpetuating these symptoms. Peripheral pro‐inflammatory cytokines, such as IL‐6 and TNF‐α, can access the central nervous system (CNS) through compromised BBB integrity or via signaling through afferent neural pathways like the vagus nerve. Once within the CNS milieu, these cytokines can activate microglia—the resident immune cells of the brain—leading to a state of microglial priming. In this sensitized state, microglia exhibit exaggerated responses to minor stimuli, contributing to chronic neuroinflammation. Neuroimaging and cerebrospinal fluid studies in PASC patients have demonstrated markers of glial activation and elevated neuroinflammatory mediators. Therapeutically, this insight supports the exploration of CNS‐penetrant anti‐inflammatory agents, such as minocycline or low‐dose naltrexone, as well as neuromodulatory techniques like transcutaneous vagal nerve stimulation, which may dampen systemic inflammation and rebalance neuroimmune interactions [20, 21].

## 9 Role of Endothelial Cytokine Signaling and Microvascular Dysfunction

The endothelium plays a central role in orchestrating vascular homeostasis, and its dysfunction is increasingly recognized as a hallmark of both acute COVID‐19 and its chronic sequelae. Pro‐inflammatory cytokines such as IL‐1β, IL‐6, and TNF‐α exert potent effects on endothelial cells, inducing a pro‐thrombotic and pro‐adhesive state that contributes to microvascular inflammation. In PASC, persistent endothelial activation may underlie a spectrum of symptoms, including exercise intolerance, autonomic dysregulation, and chronic fatigue. Capillary rarefaction, endothelial senescence, and impaired nitric oxide signaling have all been observed in long COVID patients, pointing to a state of ongoing vascular stress. Therapeutic strategies aimed at restoring endothelial integrity are gaining attention. These include statins for their pleiotropic anti‐inflammatory and endothelial‐protective effects, as well as agents like ACE inhibitors and Tie2 receptor agonists that enhance endothelial repair and barrier function. Targeting endothelial cytokine signaling could not only alleviate systemic inflammation but also address the vascular underpinnings of persistent PASC symptoms [22, 23].

## 10 Cytokine Network Disruption Through Systems Biology

While early therapeutic efforts focused on individual cytokines, systems biology has revealed that the inflammatory response in PASC operates through complex, interconnected cytokine networks. Disruption of a single cytokine may not sufficiently dampen the entire cascade due to compensatory feedback mechanisms. Computational modeling and network analysis have identified key regulatory hubs—such as the IL‐6–STAT3–SOCS3 axis or the TNF–NF‐κB loop—that act as amplifiers within the cytokine storm. Intervening at these nodal points may yield broader and more sustained anti‐inflammatory effects. Moreover, systems biology approaches have been instrumental in predicting synergistic effects of combination therapies and identifying patient‐specific network vulnerabilities. Multitarget kinase inhibitors, which simultaneously inhibit multiple signaling cascades, and combination regimens tailored to individual cytokine profiles represent the next frontier in personalized anti‐inflammatory therapy. These strategies are not only mechanistically rational but may also circumvent the pitfalls of targeting single mediators in a redundant cytokine milieu [24, 25].

## 11 Chronic COVID Biologics and Monoclonal Antibody Repurposing

The rapid development and deployment of monoclonal antibodies (mAbs) during the acute phase of the COVID‐19 pandemic have created a therapeutic armamentarium that may be repurposed for PASC. Agents targeting IL‐6 (e.g., tocilizumab), IL‐1β (e.g., anakinra), and TNF‐α (e.g., infliximab) have well‐characterized safety profiles and mechanisms of action. While initially used in severe COVID‐19 pneumonia, these biologics are now being evaluated for their utility in long COVID, particularly in patients with biomarker‐confirmed cytokine elevations. Emerging biologics, such as satralizumab (a next‐generation anti–IL‐6 receptor antibody) and CCR5 antagonists like leronlimab, are also under consideration for their potential to disrupt chronic immune cell trafficking and cytokine release. Additionally, trials exploring anti‐CXCL10 and anti‐IFN‐γ mAbs are underway, reflecting a shift toward targeting chemokine‐mediated immune dysregulation. The repurposing of these agents for long COVID represents a promising translational strategy, especially when guided by cytokine profiling and immunophenotyping [26, 27].

## 12 Proposed Mechanisms of Persistent Cytokine Dysregulation in PASC

Persistent immune activation and low‐grade inflammation are emerging as central contributors to the heterogeneous manifestations of PASC. While a definitive causal pathway has not been established, multiple mechanisms have been proposed to explain why cytokine dysregulation may persist long after acute SARS‐CoV‐2 infection.

### 12.1 Residual Viral Antigens and Persistent Immune Stimulation

One potential driver of sustained cytokine activity is the presence of residual viral RNA or protein fragments in tissues. These remnants, even in the absence of replication‐competent virus, may continue to stimulate innate and adaptive immune responses. For example, viral antigens within cardiac or pulmonary tissue could trigger localized inflammatory cascades, promoting ongoing production of IL‐6, TNF‐α, and other proinflammatory cytokines. This mechanism aligns with observations of tissue‐specific inflammatory signatures in post‐COVID autopsy and biopsy studies, suggesting that residual viral components act as persistent immunological stimuli [28, 29].

### 12.2 Immune Cell Dysfunction and Dysregulated Signaling

Persistent cytokine elevation may also reflect long‐term alterations in immune cell function. Dysregulated T‐cell responses, impaired regulatory T‐cell activity, and monocyte/macrophage hyperactivation have been documented in patients with prolonged post‐COVID symptoms. Such dysfunction can perpetuate a cycle of cytokine production, tissue injury, and secondary immune activation. Single‐cell and omics analyses reveal altered transcriptional and signaling profiles in immune cells, supporting the concept that cellular‐level dysregulation underlies the chronic inflammatory state observed in PASC [29, 30].

### 12.3 Endothelial Activation and Vascular Inflammation

Endothelial cells appear particularly vulnerable to post‐COVID injury. Persistent endothelial activation can promote microvascular inflammation, leukocyte recruitment, and prothrombotic states, all of which amplify systemic cytokine signaling. This mechanism may contribute to organ‐specific sequelae such as pulmonary fibrosis, myocardial injury, and neurologic deficits, reflecting the interplay between vascular inflammation and immune dysregulation [31, 32].

### 12.4 Autoimmunity and Aberrant Antibody Responses

Autoantibody production and molecular mimicry represent additional mechanisms driving prolonged inflammation. SARS‐CoV‐2 infection has been associated with the generation of autoantibodies that target self‐antigens, potentially sustaining immune activation in the absence of viral persistence. Autoimmune‐mediated cytokine production may explain some of the neurological, cardiovascular, and musculoskeletal manifestations of PASC, particularly in patients lacking detectable viral RNA [33, 34].

## 13 Competing Hypotheses: Viral Persistence Versus Autoantibody‐Mediated Inflammation

These mechanisms are not mutually exclusive and may operate simultaneously. In some individuals, residual viral antigens may drive chronic immune stimulation, whereas in others, autoantibody‐mediated inflammation predominates. The relative contribution of these pathways likely varies across PASC phenotypes and organ systems, explaining the observed heterogeneity in symptomatology. Integrating molecular profiling, immune phenotyping, and tissue‐specific analyses will be crucial to delineate the dominant mechanisms in individual patients.

## 14 Molecular and Mechanistic Insights Into Cytokine Dysregulation in PASC

Emerging molecular and omics‐based studies have begun to elucidate the mechanistic underpinnings of persistent inflammation in PASC, revealing pathway‐level perturbations, organ‐specific effects, and immune cell dysregulation that may sustain cytokine activity. These insights help bridge clinical observations of prolonged symptoms with underlying molecular mechanisms. Transcriptomic analyses of post‐COVID heart tissue indicate aberrant activation of the TNF‐NF‐κB signaling pathway in cardiomyocytes [33]. This pathway is a central regulator of inflammation, driving the expression of proinflammatory cytokines such as IL‐6, IL‐1β, and TNF‐α. Its inappropriate activation in the post‐viral setting may contribute to persistent myocardial inflammation, fibrosis, and cardiac dysfunction observed in some PASC patients. Beyond cardiomyocytes, NF‐κB signaling has been implicated in endothelial cells and immune subsets, suggesting systemic consequences of pathway dysregulation.

Multi‐omics studies of post‐COVID patients reveal significant mitochondrial perturbations, including impaired electron transport, increased reactive oxygen species, and altered mitochondrial biogenesis [34]. These disruptions can sensitize immune cells to hyperactivation, amplify cytokine production, and promote cellular apoptosis in affected tissues. Moreover, mitochondrial dysfunction in neural and endothelial cells may underlie neurological symptoms and contribute to BBB compromise, linking metabolic dysregulation to systemic inflammatory signaling. Omics‐based investigations highlight compromised BBB integrity as a mechanism connecting peripheral immune dysregulation to CNS manifestations of PASC [34]. Cytokines such as IL‐6 and TNF‐α, produced peripherally, may cross or signal through a disrupted BBB, triggering neuroinflammatory cascades that contribute to cognitive impairment, fatigue, and mood disturbances. Endothelial dysfunction within the CNS vasculature amplifies local immune activation, reinforcing a cycle of neuroinflammation.

Single‐cell RNA sequencing and proteomic analyses reveal persistent alterations in immune cell populations, including activated monocytes, dysregulated T cells, and reduced regulatory T‐cell function. These cellular perturbations reinforce chronic cytokine production and may interact with tissue‐specific inflammatory pathways. For example, monocyte‐derived cytokines can activate endothelial cells, further amplifying TNF‐NF‐κB signaling, while T‐cell dysfunction may impair the resolution of inflammation [33]. These data suggest that persistent cytokine dysregulation in PASC is mediated by a network of interdependent molecular pathways. TNF‐NF‐κB signaling, mitochondrial dysfunction, and endothelial activation interact with dysregulated immune cell responses to sustain a proinflammatory milieu. Importantly, these pathways exhibit organ‐specific expression patterns, which may explain the heterogeneity of PASC phenotypes, including cardiovascular, neurological, and pulmonary manifestations [34].

### 14.1 Therapeutic Strategies Targeting Cytokine Dysregulation in PASC

Management of PASC remains a major clinical challenge, in part due to the heterogeneity of symptoms and the incomplete understanding of underlying mechanisms. Given the emerging evidence that persistent cytokine dysregulation contributes to organ‐specific and systemic pathology, therapeutic strategies aimed at modulating residual inflammatory activity are under active investigation. These strategies range from targeted cytokine inhibition to broader immunomodulatory and supportive interventions [5, 10]. Therapies that directly inhibit specific proinflammatory cytokines or their signaling pathways offer a rational approach for patients in whom persistent cytokine activity is prominent. Monoclonal antibodies such as tocilizumab have demonstrated efficacy in reducing IL‐6–driven inflammation in acute COVID‐19. Emerging data suggest that in selected PASC patients, particularly those with persistent fatigue, cardiopulmonary inflammation, or systemic inflammatory biomarkers, IL‐6 blockade may mitigate chronic inflammation [14].

Mechanistically, IL‐6 inhibition can dampen downstream NF‐κB signaling, interrupting a key axis of tissue injury. Aberrant activation of the TNF‐NF‐κB pathway, particularly in cardiac and endothelial tissues, provides a rationale for TNF‐targeted therapy. Although evidence in PASC is still limited, selective TNF inhibition could potentially reduce ongoing myocardial and vascular inflammation. Small‐molecule inhibitors of the JAK‐STAT signaling pathway, including baricitinib, can attenuate cytokine signaling more broadly. By targeting multiple cytokine pathways simultaneously, JAK inhibitors may be particularly beneficial in patients with overlapping immune dysregulation and multi‐organ involvement [35]. Short‐term corticosteroid therapy can reduce systemic inflammation and endothelial activation. Their use in PASC should be carefully balanced against potential side effects, particularly in patients with metabolic or cardiovascular vulnerability. With anti‐inflammatory and microtubule‐stabilizing effects, colchicine may attenuate endothelial activation and leukocyte recruitment, providing a mechanistic link to vascular inflammation observed in PASC [36].

Structured exercise and pulmonary rehabilitation programs can improve fatigue, cardiorespiratory function, and quality of life while indirectly modulating low‐grade inflammation. Nutraceuticals such as omega‐3 fatty acids, vitamin D, and antioxidants may provide mild anti‐inflammatory effects and support mitochondrial function, addressing mechanisms implicated in post‐COVID tissue dysfunction. Techniques including graded activity, biofeedback, and cognitive rehabilitation may help modulate neuroimmune interactions, particularly in patients with persistent cognitive and neurological symptoms linked to BBB disruption and neuroinflammation [37]. Identifying patient‐specific cytokine signatures can guide targeted intervention, ensuring that therapies such as IL‐6 or TNF inhibitors are administered to those most likely to benefit. Integrating molecular and omics data, including pathway‐level activation in cardiac, neural, or pulmonary tissues, can inform focused interventions, minimizing systemic immunosuppression while addressing the root inflammatory drivers. Given the interplay between immune dysregulation, endothelial activation, and mitochondrial dysfunction, combined therapies targeting multiple pathways may offer synergistic benefit in selected patients [38].

## 15 Immunometabolism as a Therapeutic Interface

The metabolic state of immune cells profoundly influences their function and fate. In the context of PASC, immune cells—particularly monocytes, macrophages, and T cells—exhibit metabolic reprogramming characterized by increased glycolysis and mitochondrial dysfunction. This metabolic phenotype supports the sustained production of inflammatory cytokines and resists resolution of inflammation. Immunometabolism thus emerges as a critical interface between immune activation and cellular energy regulation. Agents that modulate metabolic pathways are under investigation for their potential to shift immune cells from a pro‐inflammatory (M1‐like) to an anti‐inflammatory (M2‐like) state. Metformin, known for its activation of AMP‐activated protein kinase (AMPK), has shown promise in reducing systemic inflammation and improving metabolic flexibility in immune cells. Other candidates, such as 2‐deoxy‐d‐glucose (2‐DG) and peroxisome proliferator‐activated receptor gamma agonists, are being explored for their ability to modulate glycolytic flux and mitochondrial biogenesis. These metabolic interventions may offer a novel, indirect route to temper the cytokine storm in long COVID without traditional immunosuppression [22, 23].

## 16 Biomarker‐Guided Precision Medicine

The heterogeneity of PASC and the complex nature of cytokine dysregulation necessitate a personalized approach to therapy. Biomarker‐guided precision medicine offers a promising strategy to optimize immunomodulatory treatment by identifying patients with active cytokine storms and tailoring interventions to their specific inflammatory profiles [24]. Key inflammatory biomarkers such as IL‐6, CRP, ferritin, d‐dimer, and soluble IL‐2 receptor (sIL‐2R) have been studied extensively in acute COVID‐19 and are now being evaluated in PASC. Elevated levels of these markers can indicate ongoing immune activation and help stratify patients based on disease severity and risk of progression. In addition, profiling cytokine panels using multiplex assays or next‐generation sequencing can provide a comprehensive snapshot of the immune milieu, guiding the choice of targeted therapies such as IL‐6 inhibitors or JAK inhibitors [25].

Beyond circulating cytokines, advances in immunophenotyping allow for characterization of immune cell subsets involved in PASC, including exhausted T cells, hyperactivated macrophages, and dysregulated B cells. Integration of these cellular biomarkers with clinical parameters enhances the predictive accuracy for therapeutic response and disease trajectory. Moreover, emerging “omics” technologies—transcriptomics, proteomics, and metabolomics—offer deeper insights into molecular pathways driving persistent inflammation, potentially unveiling novel therapeutic targets [26, 27]. Implementing biomarker‐guided approaches also facilitates dynamic monitoring of treatment efficacy and adverse effects, enabling timely adjustments to therapy. For example, serial measurement of inflammatory markers can inform tapering of immunosuppressive drugs or escalation to combination regimens. This iterative process minimizes overtreatment and reduces the risk of complications such as secondary infections or immunoparesis [39].

## 17 Conclusion

PASC represents a complex and evolving clinical challenge, encompassing a wide spectrum of organ‐specific and systemic complications. Accumulating evidence indicates that persistent immune dysregulation and low‐grade inflammation, including elevated cytokine activity, play a significant role in the pathogenesis of PASC in certain patients, contributing to ongoing tissue injury and functional impairment. However, the heterogeneity of clinical presentations, variability in cytokine profiles, and incomplete mechanistic understanding highlight that the precise causal role of a sustained cytokine storm remains under investigation. Molecular and omics‐based studies have begun to elucidate organ‐specific pathways, such as TNF‐NF‐κB activation in cardiomyocytes and mitochondrial dysfunction with BBB compromise, which may link cytokine dysregulation to downstream pathology. These insights underscore the importance of precision‐based, mechanistic approaches to identifying therapeutic targets. Current and emerging strategies, ranging from cytokine‐targeted biologics to immunomodulators and supportive interventions, show promise, yet rigorous, PASC‐specific clinical trials are required to establish efficacy and safety.

## Author Contributions

**Emmanuel Ifeanyi Obeagu:** conceptualization, methodology, validation, writing – original draft, writing – review and editing, supervision.

## Funding

The author received no specific funding for this work.

## Conflicts of Interest

The author declares no conflicts of interest.

## Transparency Statement

The lead author Emmanuel Ifeanyi Obeagu affirms that this manuscript is an honest, accurate, and transparent account of the study being reported; that no important aspects of the study have been omitted; and that any discrepancies from the study as planned (and, if relevant, registered) have been explained.

## Data Availability

The author has nothing to report.
